# Supplementary material for: Chromothripsis during telomere crisis is independent of NHEJ, and consistent with a replicative origin
Source: Genome Res. 2019 May;29(5):737–49. doi: 10.1101/gr.240705.118 (PMC6499312; doi:10.1101/gr.240705.118)
Supplement: Supplemental Material [file supp_gr.240705.118_Supplemental_file_1.zip › contigs/annotated_contigs/DB51/contig.3.DB51_length_690_mean_cov_8.5.docx]

**DB51_length_690_mean_cov_8.5**

```
ACTGTGTCCTCTCATGGTGGAAGGATTAATGAACTTCTATGGGCCTCTTGTATAAGGGCACTAATCCTATGTAGGAAGGCTCTGCCTTC
 >chr6:70531908-70532253 - E=8e-196
ATGGCCTAACAACCTCCCCAAAGGTCCCACCTCCTGATACCATCAGCTTGGGGTTTAGGATTTCAACATATACATTTGAAAGGGACATA

TGCACTAAGACTATAGTACCCTATATGGGTAAATCTTTAAATATTACTAAACTATGTAAAAGTACACTTGAAACTTGAGTACATAGAAA

CATCTTATGTCTATGTAATTATCAAAATGTTTTATTTTCTGGAGTTAATTTTAAGTCAAGTCAATTTTAAAATTTA|CA|GGGTGATTA
                                                                             >chr18:551
GTATATCTTTGCTTTTGCAGTGACCACCAAAATCATCCTGGAAAGACCTCTGGGAGAGATGGCAGAGCTTGATCCACTCTGCATGCCAA
84923-55185028 - E=2e-51 p=4e-02
GCT|GG|GCATGGTGGCGTGCACCTGTAATCCCAGCTACTCAGGAGGCTGAGACAGGAGAATCGCTTGAACCTGGAAGGTGGAGGTTGC
    >chr10:31296184-31296371 - E=4e-82
AGTGAGCTGAGATCACGCCACTGCACTTCAGCCAGGGCAACAGTGTGAGACTCTGTCTCCAAAAAAAACAAACACCAAAAAAACCTTTG

TGAC|GTAAACCCACCCAAATGTCTTATCTCCCCTTCTGGATGTCGGGGGGGGAAACTGCTGTTTTGTTCTT
```
